# Investigating diagnosis, treatment, and burden of disease in patients with ankylosing spondylitis in Central Eastern Europe and the United States: a real-world study

**DOI:** 10.1007/s10067-021-05864-8

**Published:** 2021-07-28

**Authors:** T. Korotaeva, O. Dina, E. Holdsworth, L. Fallon, G. Milligan, S. Meakin, L. Wang, R. Vasilescu, J. C. Cappelleri, A. Deodhar

**Affiliations:** 1 Institute of Rheumatology V.A. Nasonova, 115522 Kashirskoe shosse 34-A, Moscow, Russia; 2 Pfizer Inc, New York, NY USA; 3 Adelphi Real World, Bollington, UK; 4 Pfizer Inc, Kirkland, QC Canada; 5 Pfizer Inc, Groton, CT USA; 6 Pfizer, Brussels, Belgium; 7 Oregon Health & Science University, Portland, OR USA

**Keywords:** Ankylosing spondylitis, Diagnosis, Disease burden, Quality of life, Treatment

## Abstract

**Introduction/Objectives:**

Ankylosing spondylitis (AS) is a chronic inflammatory immune-mediated condition. We compared AS diagnosis, treatment, and burden in Central Eastern European countries (CEE), where this has been less researched, and the United States (US) from a real-world perspective.

**Methods:**

Point-in-time survey of rheumatologists and their AS patients was conducted in the US (Apr–Oct 2018) and CEE (Aug–Nov 2019) via physician- and patient-completed record forms, including clinical and patient-reported outcomes. Statistical analysis included descriptive statistics, *t*-tests, Fisher’s exact tests, and generalized linear models.

**Results:**

In total, 487 patients were recruited from 88 rheumatologists in the US and 922 patients from 126 rheumatologists in CEE. Time from onset of symptoms to final AS diagnosis was longer in CEE than the US (4.2 vs 2.7 years, *p* < 0.05). At diagnosis, a greater use of conventional synthetic disease-modifying antirheumatic drugs (DMARDs) and injected steroids was reported in CEE vs the US (43.7% vs 27.6%, *p* < 0.05; 19.3% vs 8.7%, *p* < 0.05). 22.9% of US patients received a biologic DMARD at diagnosis vs 10% of CEE patients (*p* < 0.05). At current consultation, biologic DMARD use in CEE was lower vs the US (27.9% vs 71.0%, *p* < 0.05). CEE vs US patients had greater disease activity (mean Bath Ankylosing Spondylitis Disease Activity Index 4.2 vs 3.1, *p* < 0.05) and worse quality of life (QoL; mean Ankylosing Spondylitis Quality of Life Questionnaire score 6.2 vs 8.4, *p* < 0.05).

**Conclusions:**

AS patients in CEE vs the US faced slower diagnosis and worse access to biologics, disease activity, and QoL. Whether early access to biologics can improve symptoms, QoL, and daily activities in AS patients in CEE remains to be seen.

**Table:** 

**Key Points** • *The study provided evidence on the real-world approach to the diagnosis, treatment, and burden of axSpA (axial spondyloarthritis) in CEE compared with the US.* • *The study reported patients in CEE experienced longer delays in diagnosis and poorer access to biologics than in the US.* • *This may have resulted in higher disease activity, greater levels of pain, and poorer outcomes, as reported by patients with axSpA in CEE.*

**Supplementary Information:**

The online version contains supplementary material available at 10.1007/s10067-021-05864-8.

## Introduction

Axial spondyloarthritis (axSpA) is a chronic inflammatory immune-mediated condition that predominantly affects the axial skeleton. The main symptom is chronic, inflammatory lower back pain, but symptoms can also include inflammatory peripheral arthritis, enthesitis, and extra-musculoskeletal manifestations such as psoriasis, inflammatory bowel disease, and uveitis [1].

The term axSpA encompasses both ankylosing spondylitis (AS), also known as radiographic axSpA, and non-radiographic axSpA (nr-axSpA) [2]. The condition usually starts in the third decade of life with a male to female ratio of 2:1 for AS and equal gender prevalence for nr-axSpA [3]. In its advanced stage, AS leads to fusion of sacroiliac joints and the spine.

The prevalence of axSpA in the United States (US) has been estimated to be 0.9–1.4% [4], while the prevalence in the rest of the world ranges from 9 to 30 per 10,000 persons, depending on geographic area, study population, data source, or case definition. The progression of patients with nr-axSpA to AS is slow, with estimates of 5.1% in 5 years and 19% in 10 years [5]. Although patients with nr-axSpA may have shorter disease duration and lack definitive radiological changes of sacroiliitis, they demonstrate a substantial physical and social burden of illness, with self-reported disease activity and functional impairments comparable to those found in patients with AS [6].

The diagnosis of axSpA may be challenging, as no formal diagnostic criteria are available. The recent classification criteria for axSpA developed by the Assessment of Spondyloarthritis International Society (ASAS) include a combination of features such as sacroiliitis on either conventional radiography or magnetic resonance imaging (MRI), presence of human leucocyte antigen B27 (HLA-B27), raised C-reactive protein, and other associated clinical characteristics [7]. With only 37% of patients with AS in the US diagnosed by rheumatologists [8], time to diagnosis has been reported to be as long as 14 years in some patients, indicating the potential failure to recognise the condition by non-rheumatologists [9].

Delayed diagnosis is associated with more functional impairment, higher healthcare costs, and worse quality of life and work productivity outcomes in patients with AS [10, 11]. Early diagnosis results in patients receiving therapy sooner where it is considered most effective, which should lead to reduced burdens and improved outcomes [12].

The goals of treatment are to alleviate symptoms, improve functioning, maintain the ability to work, decrease disease complications, and avoid skeletal damage as much as possible [13]. Current ASAS-European League Against Rheumatism (EULAR) treatment guidelines for axSpA recommend continuous treatment with nonsteroidal anti-inflammatory drugs (NSAIDs) as a first-choice therapy. In patients who do not improve with continuous NSAID treatment, biological disease-modifying antirheumatic drugs (bDMARDs), including tumour necrosis factor inhibitors (TNFi) and interleukin-17 (IL-17) inhibitors (e.g. secukinumab and ixekizumab), are recommended for patients with high disease activity despite the use (or intolerance/contraindication) of at least two NSAIDs [14]. Conventional synthetic disease-modifying antirheumatic drugs (csDMARDs), such as sulfasalazine, should be considered for axSpA patients with peripheral involvement, or when a TNFi or IL-17 is not available or appropriate [14].

In the US, the 2019 guidelines from the American College of Rheumatology, Spondylitis Association of America, and the Spondyloarthritis Research and Treatment Network for the treatment of AS and nr-axSpA also recommend TNFi over an IL-17 inhibitor as the first bDMARD to be used, while secukinumab or ixekizumab is conditionally recommended over the use of a second TNFi in patients with primary nonresponse to the first TNFi [13]. Either a TNFi or an IL-17 inhibitor is favoured over the Janus kinase inhibitor, tofacitinib [13].

The diagnosis, treatment, and burden of AS have been less researched in Central Eastern European countries (CEE) than in the US [15, 16]. Given the increasing importance of these emerging markets, with recent trials recruiting a majority of patients from this region, the aim of this study was to compare the treatment journey of patients with AS, including diagnosis, treatment patterns, and burden of disease in both the CEE and the US from a real-world perspective.

## Materials and methods

### Study design

This study was an analysis of secondary data drawn from the Adelphi AxSpA Disease Specific Programme (DSP)™, a point-in-time survey of rheumatologists and their consulting patients in the US, Czech Republic, Poland, Russia, and Ukraine. DSP™s are multinational surveys collecting information on real-world clinical practice, designed to identify current disease management, and patient- and physician-reported disease impact [17].

Data were collated from a DSP™ which was conducted in the US between April and October 2018, and in CEE between August and November 2019. Data were collected through physician- and patient-completed record forms and included clinical information and patient-reported outcomes (PROs).

A geographically representative sample of physicians were recruited to participate in the DSP™ and gave informed consent to participate, with physicians recruited by local field-based interviewers using publicly available lists and screened against eligibility criteria. The data collection setting was secondary care rheumatology services (public or private hospitals, clinics, or offices). While minimal inclusion criteria governed the selection, participation was influenced by willingness to complete the survey. Following screening, physicians included in the survey were invited to complete a pre-specified patient record form (PRF) questionnaire for 3–6 consecutive patients with AS who visited for routine care. PRFs included detailed questions on patient demographics, clinical assessments, medication use, and treatment history. Patients were eligible for inclusion if aged 18 years or older, with a physician-confirmed diagnosis of AS. There were no restrictions according to treatments, clinical features such as disease activity/severity or demographics. Each patient with a physician-completed questionnaire was invited to voluntarily fill out a patient self-completed (PSC) questionnaire after providing informed consent. Patients completed their questionnaires independently from physicians, returning them in sealed envelopes to ensure confidentiality.

Data captured included physicians’ approach to AS diagnosis, tests and assessments used to confirm diagnosis, patient demographic and clinical characteristics (including the proportion of patients who were known to be HLA-B27 positive), the duration from initial symptoms to an AS diagnosis, fulfilment of classification criteria, treatment patterns (including the use of advanced treatment by AS disease activity), and patient-reported burden of disease from AS, using validated measures. Patients were invited to voluntarily complete the following validated measures of disease activity, quality of life (QoL), and general health status and productivity: the Bath Ankylosing Spondylitis Disease Activity Index (BASDAI) [18], the Ankylosing Spondylitis Quality of Life Questionnaire (ASQoL) [19], the EuroQoL 5D (EQ-5D) [20], Work Productivity and Activity Impairment (WPAI) questionnaires [21], and the ASAS Health Index (ASAS HI) [22].

### Data analysis

All PRFs were completed online to minimise the issue of missing data. If physicians did not know or have access to historical medical records, there was the option to select “don’t know” or “not applicable.” Missing data were not imputed; therefore, the base of patient data for analysis could vary from variable to variable and is reported separately for each analysis, thereby enabling the calculation of the number of missing patients. Patients were grouped according to geographical area of origin (US or CEE) and each variable of interest was reported and compared across geographical area (US vs CEE) using methods appropriate to variable type.

Physician-level data from the physician survey, patient demographics, and underlying patient condition (including age, gender, body mass index [BMI], comorbid conditions, and symptoms) from PRFs were compared using Fisher’s exact tests (for binary categorical variables) [23]. For categorical variables with more than two categories, the Fisher-Freeman-Halton test with the Mehta and Patel extension of the Fisher’s exact test was used [24, 25].

For outcomes (see Online Resource 1 for a variables list), a multivariable regression approach was used to adjust for potential confounding from age, gender, BMI, and comorbidities (using the Charlson Comorbidity Index [CCI]). In each case, a binary variable was included that represented region (US/CEE), together with the confounding variables.

For continuous outcomes, a generalized linear model with normal distribution and an identity link function was employed [26]. For categorical outcomes, a logistic regression (for the binary case) or multinomial logistic regression (for the case of more than 2 groups) was employed [27]. The output for each fitted model included means (least squares) and the *p*-values associated with CEE (compared with the US as the reference country).

For time-to-event outcomes, *t*-tests were performed. Where statistical tests were performed, *p*-values < 0.05 were considered statistically significant. Standard errors were adjusted for potential clustering within physicians [28].

All analyses used Stata Statistical Software: Release 15 (StataCorp LP, College Station, TX).

A sensitivity analysis was also conducted, which replicated the primary analysis but selected patients who were reported by physicians to have sacroiliitis identified by x-ray at diagnosis. In a real-world population of AS patients, not all patients were able to be confirmed as having sacroiliitis identified by x-ray at diagnosis. The purpose of conducting a sensitivity analysis was to, as closely as possible, match the AS population in clinical trials and also allow comment on the applicability of the results in patients with sacroiliitis identified by x-ray at diagnosis to the entire sample (see Online Resources 1–7).

### Ethical considerations

The DSP™ complies with all relevant market research guidelines and legal obligations. Data were collected according to European Pharmaceutical Marketing Research Association guidelines and thus did not require ethics committee approvals [29]. Namely, the DSP™ is non-interventional and employs solely retrospective data collection, and no identifiable protected health information was extracted during the course of the study.

## Results

### Study population

The sample included a total of 487 patients recruited from 88 rheumatologists in the US and 922 patients from 126 rheumatologists in CEE with a physician-confirmed diagnosis of AS. Patient-reported data were collected for 55% of US patients and 86% of CEE patients (US 296, CEE 793).

### Patient demographic and clinical characteristics

Key patient demographics and disease characteristics are summarised in Table 1. Patient characteristics did not differ significantly between the geographical regions in terms of age and sex, and the majority of patients were white/Caucasian in both populations. In CEE, the proportion of patients currently employed was lower than in the US, with a significantly higher proportion being smokers or ex-smokers, and those in CEE also had a lower mean BMI. Physician-reported severity of condition was similar in both regions, with over half of patients being categorised as moderate. The proportion of patients with sacroiliitis on x-ray was significantly higher in CEE relative to the US, while the key disease features of inflammatory back pain, morning stiffness for more than 30 min, enthesitis, and dactylitis at diagnosis were similar in both countries (Table 1).

**Table 1:** Patient demographic and clinical characteristics

	**US**	**CEE**	***p*-value**
**Age, years, *n***	487	922	
Mean (SD)	46.4 (14.1)	45.5 (12.7)	Ns
Median (IQR)	45.0 (36.0–56.0)	45.0 (37.0–54.0)	
**Gender, *n* (%)**	487	922	
Male	344 (70.6)	665 (72.1)	Ns
Female	143 (29.4)	257 (27.9)	
**BMI, *n***	487	922	
Mean (SD)	27.4 (4.5)	26.0 (4.1)	< 0.001
Median (IQR)	26.8 (24.6–29.4)	25.5 (23.1–28.1)	
**Ethnicity, *n* (%)**	487	922	
White/Caucasian	393 (80.7)	899 (97.5)	< 0.001
African American/Afro-Caribbean	42 (8.6)	1 (0.1)	
Native American	1 (0.2)	0 (0.0)	
Asian-Indian subcontinent	7 (1.4)	0 (0.0)	
Asian–other	8 (1.6)	0 (0.0)	
Chinese	2 (0.4)	0 (0.0)	
Hispanic/Latino	23 (4.7)	0 (0.0)	
Middle Eastern	3 (0.6)	2 (0.2)	
Mixed race	7 (1.4)	7 (0.8)	
Asian	0 (0.0)	3 (0.3)	
Other	1 (0.2)	10 (1.1)	
**Employment status, *n* (%)**	483	895	
Working full-time	342 (70.8)	486 (54.3)	< 0.001
Working part-time	32 (6.6)	125 (14.0)	
On long-term sick leave	5 (1.0)	38 (4.2)	
Homemaker/student/retired	81 (16.8)	189 (21.1)	
Unemployed	23 (4.8)	57 (6.4)	
**Smoking status, *n* (%)**	454	804	
Current smoker	44 (9.7)	184 (22.9)	< 0.001
Ex-smoker	99 (21.8)	221 (27.5)	
Never smoked	311 (68.5)	399 (49.6)	
**Patient severity at diagnosis, *n* (%)**^**a**^	394	832	
Mild	20 (5.1)	73 (8.8)	0.005
Moderate	233 (59.1)	438 (52.6)	
Severe	141 (35.8)	321 (38.6)	
**HLA-B27 status, *n* (%)**	487	922	
Negative or untested	158 (32.4)	297 (32.2)	Ns
HLA-B27 positive	329 (67.6)	625 (67.8)	
**Current concomitant conditions, *n* (%)**	487	922	
Depression	44 (9.0)	30 (3.3)	< 0.001
Anxiety	44 (9.0)	52 (5.6)	0.019
Rheumatoid arthritis	4 (0.8)	26 (2.8)	0.012
Psoriatic arthritis	2 (0.4)	19 (2.1)	0.018
Psoriasis	9 (1.8)	36 (3.9)	0.039
Crohn’s disease	18 (3.7)	7 (0.8)	0.001
Uveitis	31 (6.4)	53 (5.7)	Ns
**Charlson Comorbidity Index, *n***	487	922	
Mean (SD)	0.1 (0.4)	0.3 (0.9)	< 0.001
**Current disease features, *n* (%)**	487	922	
Sacroiliitis identified by x-ray	144 (29.6)	500 (54.2)	< 0.001
Sacroiliitis identified by MRI	62 (12.7)	243 (26.4)	< 0.001
Spinal fusion	67 (13.8)	137 (14.9)	Ns
Joint inflammation/stiffness (not spine)	149 (30.6)	190 (20.6)	< 0.001
IBP or spinal pain	197 (40.5)	511 (55.4)	< 0.001
Back pain for 1–3 months	9 (1.8)	60 (6.5)	< 0.001
Back pain for more than 3 months	85 (17.5)	208 (22.6)	0.025
Morning stiffness for more than 30 min	179 (36.8)	314 (34.1)	Ns
Alternating buttock pain	32 (6.6)	92 (10.0)	0.032
Dactylitis	13 (2.7)	17 (1.8)	Ns
Enthesitis	31 (6.4)	49 (5.3)	Ns
Tendonitis	42 (8.6)	21 (2.3)	< 0.001
Synovitis	29 (6.0)	25 (2.7)	0.003
**Disease features at diagnosis, *n* (%)**	914	481	
Sacroiliitis identified by x-ray	625 (68.4%)	272 (56.5%)	< 0.001
Sacroiliitis identified by MRI	384 (42.0%)	121 (25.2%)	< 0.001
Spinal fusion	157 (17.2%)	66 (13.7%)	Ns
Joint inflammation/stiffness (not spine)	356 (38.9%)	238 (49.5%)	< 0.001
IBP or spinal pain	754 (82.5%)	369 (76.7%)	0.011
Back pain for 1–3 months	103 (11.3%)	32 (6.7%)	0.006
Back pain for more than 3 months	454 (49.7%)	225 (46.8%)	Ns
Morning stiffness for more than 30 min	539 (59.0%)	304 (63.2%)	Ns
Alternating buttock pain	249 (27.2%)	80 (16.6%)	< 0.001
Dactylitis	48 (5.3%)	28 (5.8%)	Ns
Enthesitis	163 (17.8%)	82 (17.0%)	Ns
Tendonitis	71 (7.8%)	78 (16.2%)	< 0.001
Synovitis	132 (14.4%)	58 (12.1%)	Ns

^a^Severity adjudged by physician’s subjective opinion

Abbreviations: *Ns*, non-significant; *BMI*, body mass index; *CEE*, Central Eastern European countries; *HLA-B27*, human leucocyte antigen B27; *IBP*, inflammatory back pain; *IQR*, interquartile range; *MRI*, magnetic resonance imaging; *SD*, standard deviation; *US*, United States

### Duration from initial symptoms to an AS diagnosis

The time from onset of symptoms to first consultation and final diagnosis of AS was significantly longer in CEE than in the US (Table 2). Patients in both regions were mainly referred by their family practitioner or other specialist, and a diagnosis was almost always made by a rheumatologist. However, the mean duration until referral to the current rheumatologist was 13.0 months in CEE compared with 4.8 months in the US (Table 3). In both the US and CEE, reasons for a delay in diagnosis included awaiting referral to the correct healthcare professional and awaiting tests to confirm diagnosis (Table 3). In CEE, one-third of patients experienced a delay due to initial diagnosis of another condition, significantly more than in the US. Patients in both the US and CEE waited a similar length of time from start of symptoms before seeking medical advice, most commonly reporting that they waited to see if their symptoms would resolve spontaneously (Table 3). Patients in the US were significantly more concerned about the cost of treatment (Table 3) but received a bDMARD sooner than patients in CEE (mean: 2.7 vs 4.0 years after initial diagnosis, *p* = 0.004) (Table 3).

**Table 2:** Patient journey from initial symptoms to diagnosis of AS (physician-reported)

	**US**	**CEE**	***p*-value**
**Time from onset of symptoms to diagnosis (years)**			
*N*	254	788	0.002
Mean	2.7	4.2	
SD	6.0	6.6	
**Time from symptom onset to initial consultation (years)**			
*N*	226	752	0.014
Mean	1.5	2.4	
SD	4.8	4.6	
**Time from initial consultation to diagnosis (years)**			
*N*	285	776	< 0.001
Mean	0.5	1.5	
SD	1.1	4.1	

Abbreviations: *CEE*, Central Eastern European countries; *SD*, standard deviation; *US*, United States

**Table 3:** Patient journey to current rheumatologist (physician- and patient-reported)

	**US**	**CEE**	***p*-value**
***Patient journey to current rheumatologist (physician-reported)***			
**Referring physician, % (SE)**^**a**^	468	876	< 0.001
Another specialist	38.0 (2.3)	35.1 (1.6)	
Family doctor	30.7 (2.2)	32.0 (1.6)	
Other physician	2.7 (0.7)	17.7 (1.3)	
No-one	28.6 (2.6)	15.3 (1.2)	
**Diagnosing physician, % (SE)**^**a**^	460	894	Ns
Rheumatologist	91.0 (1.4)	91.6 (0.9)	
Orthopaedic surgeon	2.6 (0.8)	2.3 (0.5)	
Internal medicine	2.5 (0.8)	0.9 (0.3)	
Other	3.8 (0.9)	5.2 (0.8)	
**Time to referral from previous HCP to current rheumatologist (months), *n***	213	570	
Mean^b^	5.0	13.0	0.006
SE	2.47	1.50	
**Reason for delay, % (SE)**^**a**^	76	339	
Waiting for referral to correct HCP	30.7 (5.4)	20.6 (2.2)	Ns
Requiring test to confirm diagnosis	27.0 (5.1)	30.8 (2.5)	Ns
Awaiting test results	22.9 (4.8)	25.9 (2.4)	Ns
Complicated diagnosis	18.3 (4.5)	13.1 (1.8)	Ns
Other condition initially diagnosed	11.7 (3.7)	33.4 (2.6)	< 0.001
Another condition took precedence	1.4 (1.4)	6.4 (1.3)	Ns
Symptoms not requiring further investigation	11.6 (3.7)	13.3 (1.8)	Ns
Other	10.5 (3.6)	6.5 (1.3)	Ns
***Patient self-reported time to first consultation***			
**Time before patient visited HCP (months)**	143	606	
Mean^c^	28.7	25.6	Ns
SE	4.58	2.21	
**Reason for delay in seeking medical advice, mean % (SD)**	269	802	
Waited for symptoms to resolve unaided	54.5 (3.1)	48.1 (1.8)	Ns
Tried to treat the symptoms myself first	28.8 (2.8)	31.7 (1.7)	Ns
Worried about the diagnosis	10.4 (1.9)	9.7 (1.1)	Ns
Worried about the cost of treatment	10.2 (2.0)	2.9 (0.6)	< 0.001
Did not think it was anything serious	30.8 (2.9)	28.2 (1.6)	Ns
Thought it was temporary lower back pain	32.4 (2.9)	39.3 (1.7)	Ns
Other reason	7.5 (1.7)	7.5 (0.9)	Ns
**Time from diagnosis to first bDMARD (years)**			
*N*	289	261	
Mean	2.7	4.0	0.004
SD	4.9	5.8	

^a^Least square means (percentages), standard errors, and *p*-values derived from logistic regressions with additional covariates: age, sex, BMI, and Charlson Comorbidity Index

^b^Predicted means, standard errors, and *p*-values are from an ordinary least squares regression with additional covariates: age, sex, BMI, and Charlson Comorbidity Index

^c^Least square means, standard errors, and *p*-values are from an ordinary least squares regression with additional covariates: age, sex, BMI, and Charlson Comorbidity Index

Abbreviations: *Ns*, non-significant; *bDMARD*, biological disease-modifying antirheumatic drug; *BMI*, body mass index; *CEE*, Central Eastern European countries; *HCP*, healthcare professional; *SD*, standard deviation; *SE*, standard error; *US*, United States

### Treatment patterns

At diagnosis, a higher use of csDMARDs and injected steroids was reported in CEE compared with the US. Almost a quarter of patients in the US received a bDMARD at diagnosis, compared to 10% in CEE (Table 4). At the time of the current study, almost three-quarters of patients in the US were receiving a bDMARD, with levels of bDMARD use in CEE significantly lower than the US (Table 4), although the mean number of bDMARD treatments per patient was similar in both the US and CEE (mean [SD] 1.3 [0.6] and 1.2 [0.6], respectively). The number of csDMARDs initiated before a bDMARD was similar in both the US and CEE (mean [SD] 1.3 [0.6] and 1.2 [0.4], respectively), with sulfasalazine being prescribed in significantly more patients in CEE than the US (79.3% and 49.4%, respectively; *p* < 0.001). Methotrexate was preferred over sulfasalazine in 62.7% of patients in the US, but in only 50.6% of patients in CEE.

**Table 4:** Treatment patterns

	**US**	**CEE**	***p*-value**
**Treatments prescribed at diagnosis, % (SE)**^a^	487	922	
NSAID	74.6 (2.0)	76.9 (1.4)	Ns
Non-opioid analgesic	10.8 (1.4)	16.0 (1.2)	0.001
Opioid analgesic	8.5 (1.3)	3.8 (0.6)	< 0.001
Oral corticosteroid	17.4 (1.8)	13.3 (1.1)	0.046
Injected corticosteroid	8.7 (1.3)	19.3 (1.3)	< 0.001
csDMARD	27.6 (2.1)	43.7 (1.7)	< 0.001
bDMARD	22.9 (1.9)	10.2 (1.0)	< 0.001
None of the above	1.2 (0.5)	1.4 (0.4)	Ns
Did not know	8.9 (1.3)	4.1 (0.7)	< 0.001
**Treatments currently prescribed, % (SE)**^**a**^	487	922	
NSAID	39.5 (2.3)	59.9 (1.6)	< 0.001
Non-opioid analgesic	4.5 (1.0)	9.7 (1.0)	< 0.001
Opioid analgesic	7.3 (1.2)	3.0 (0.6)	< 0.001
Oral corticosteroid	5.4 (1.1)	9.8 (1.0)	0.007
Injected corticosteroid	5.3 (1.0)	6.9 (0.8)	Ns
csDMARD	22.3 (1.9)	41.1 (1.6)	< 0.001
bDMARD	71.0 (2.1)	27.9 (1.5)	< 0.001
None of the above	1.9 (0.7)	2.1 (0.5)	Ns

^a^Least square means (percentages), standard errors, and *p*-values are from logistic regressions with additional covariates: age, sex, BMI, and Charlson Comorbidity Index

Abbreviations: *Ns*, non-significant; *bDMARD*, biological disease-modifying antirheumatic drug; *BMI*, body mass index; *CEE*, Central Eastern European countries; *csDMARD*, conventional synthetic disease-modifying antirheumatic drug; *HCP*, healthcare professional; *NSAID*, nonsteroidal anti-inflammatory drug; *SE*, standard error; *US*, United States

### Patient-reported outcomes

**Fig. 1:**
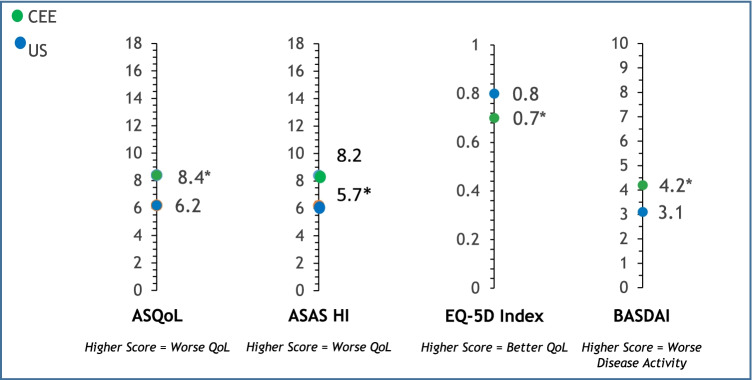
ASQoL, ASAS HI, EQ-5D, and BASDAI mean scores. Least square means and *p*-values are from an ordinary least squares regression with additional covariates: age, sex, BMI, and Charlson Comorbidity Index. *p-value CEE vs US < 0.05. Abbreviations: ASAS HI, Assessment of Spondyloarthritis International Society Health Index; ASQoL, Ankylosing Spondylitis Quality of Life Questionnaire; BASDAI, Bath Ankylosing Spondylitis Disease Activity Index; BMI, body mass index; CEE, Central Eastern European countries; EQ-5D, EuroQoL 5D; QoL, quality of life; SE, standard error; US, United States

**Fig. 2:**
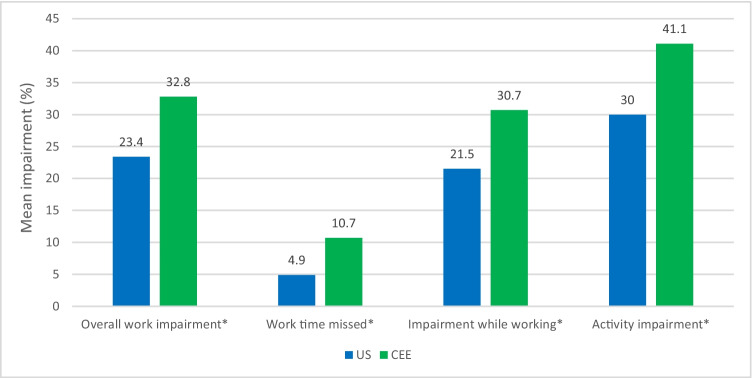
Work productivity and activity impairment. Estimated means and *p*-values are from an ordinary least squares regression with additional covariates: age, sex, BMI, and Charlson Comorbidity Index. *p-value CEE vs US < 0.05. Abbreviations: CEE, Central Eastern European countries; US, United States

Disease activity (BASDAI) was greater in patients in CEE vs US (4.2 vs 3.1, respectively, *p* < 0.05). In the US, 68.8% of patients had a BASDAI score of 4 or less, while in CEE, it was 48.2% (*p* < 0.05). Patients in the US had a lower score (indicating better QoL) on the ASQoL than those in CEE (6.2 vs 8.4, respectively, *p* < 0.05). In the US, 65.0% of patients had an ASQoL score of 8 or less, while in CEE, it was 50.7% (*p* < 0.05). A significantly lower mean EQ-5D index for the CEE population was observed relative to the US (0.7 vs 0.8, respectively, *p* < 0.05)*.* The mean ASAS HI score for the US population indicated a moderate impairment in functioning, while patients in CEE had significantly greater and more severe impairment (5.7 vs 8.2, respectively, *p* < 0.05). In the US, 39.3% of patients had a score of less than 4; 31.4% had a score of 4–8; and 29.3% had a score greater than 8 (*p* < 0.05). In CEE, these proportions were 21.0%, 29.9%, and 49.1%, respectively (*p* < 0.05). Patients in CEE also had a significantly higher mean pain score than those in the US (4.3 vs 3.5, respectively, *p* < 0.05) (means in Fig. 1, proportions not shown). Impairment in work productivity was significantly higher among patients in CEE than in the US (33% vs 23%, respectively, *p* < 0.05)*.* Activity impairment was also significantly higher in CEE than in the US (41% vs 30%, respectively, *p* < 0.05) (Fig. 2).

### Sensitivity analysis

A sensitivity analysis that replicated the primary analysis found that the results in patients who were confirmed to have sacroiliitis on x-ray at diagnosis (US *n* = 272, 56.7%; CEE *n* = 625, 68.4%) were highly consistent with the overall sample results in terms of demographics, clinical status, treatments prescribed, journey to patient diagnosis, and patient-reported outcomes (see Online Resources 2–7). Minor differences included physician-perceived severity at diagnosis and prevalence of a positive HLA-B27 result, with patients in the sensitivity analysis more frequently being perceived as “severe” (overall analysis—US: 35.8% and CEE: 38.6% severe; sensitivity analysis—US: 40.3% and CEE: 42.5% severe), and more frequently being positive for HLA-B27 (overall analysis—US: 67.6% and CEE: 67.8% positive; sensitivity analysis—US: 77.2% and CEE: 74.7% positive). Treatment patterns between the two analyses were also consistent, with minor difference including more bDMARD prescribed at both diagnosis and currently in the sensitivity analysis population (overall analysis bDMARD at diagnosis—US: 22.9% and CEE: 10.2%; sensitivity analysis bDMARD at diagnosis—US: 30.1% and CEE: 12.2%; overall analysis bDMARD currently—US: 71.0% and CEE: 27.9%; sensitivity analysis bDMARD currently—US: 75.9% and CEE: 31.9%).

## Discussion

Relatively few studies have compared clinical outcomes in patients with AS from different geographical regions [15, 16] and this study of patients with AS is novel in comparing diagnostic procedures, treatment patterns, and outcomes in the US and CEE from a real-world perspective. These regions were selected in large part due to their heterogeneity and their importance as emerging markets. Another factor was the ability to recruit an optimal number of physicians and patients to establish a robust dataset for the analyses. This will allow for a more thorough interpretation of trial results, given that recent trials for AS and nr-axSpA are recruiting most patients from the CEE region. We also wanted to understand whether any similarities or differences in these populations could lead to any differences in treatment effect across regions, given new drug approvals typically occur in the US first.

Overall, the study shows that treatment patterns are very different, with reported higher use of csDMARDs and injected corticosteroids at diagnosis, and lower levels of bDMARD use reported at both diagnosis and at the current consultation in the CEE than in the US, potentially indicating a lack of access to treatment and different approach to care in the CEE region.

It is well documented that patients with AS can experience delays in diagnosis [10], and evidence from studies across the world indicates that diagnostic delay may extend over several years [8, 30]. In the CEE, the referral time from the initial healthcare professional to rheumatologist was significantly longer than in the US, suggesting that the primary care system in CEE is a potential source of delays. The time to diagnosis was also found to take longer in CEE than in the US. Also, patients in CEE reported poorer QoL, greater work and activity impairment, more work time missed, and higher disease activity than patients in the US.

Two-thirds of patients in both the US and CEE were reported to be HLA-B27 positive, which is lower than other studies that have suggested the prevalence of HLA-B27 in patients with AS to be as high as 80–94% [31–33]. Since the data collected in this real-world study employed a non-interventional approach, data was only available if a positive result was recorded in medical records, we were not able to ascertain if more patients were tested and proved HLA-B27 negative or were untested. It is feasible that patients in this sample had not been tested for HLA-B27 during their routine care, leading to underreporting or underrepresenting of positive HLA-B27 status.

After failure of NSAIDs, biologic DMARDs are the only proven efficacious therapies for the treatment of axSpA, with the suppression of inflammation shown to improve and maintain QoL in patients [34]. However, due to the high cost of biologics, considerable differences in their utilization exist, with many countries restricting access despite professional society guideline recommendations. The adoption of biologics by healthcare providers has been reported to be less in many CEE countries [35, 36], and differences in utilization have been reported across medical specialties, healthcare providers, and at a regional and national level [37].

In our real-world study, over three-quarters of patients (72.1%) with AS in the US received bDMARDs compared with approximately one-quarter of patients (27.3%) in CEE. It has been previously reported that access to such medication may be limited in CEE countries due to reimbursement systems only covering low cost treatments, with a lack of access to rheumatological care in particular [38].

In terms of PROs, we found that the mean BASDAI was higher in patients in CEE (mean score 4.2 vs 3.1 in US). The patient-acceptable symptom state (PASS) is estimated as a score of 4.1 [39], which a significantly greater proportion of patients in the US had achieved (68.8% vs 48.2% in CEE). Patients in the US vs CEE also had a significantly lower mean ASQoL score (6.2 vs 8.4). The PASS for ASQoL has been calculated at < 8.0 [40], which a significantly greater proportion of patients in the US had achieved (65.0% vs 50.7% in CEE), indicating a higher proportion of patients experience an acceptable quality of life in the US. The mean EQ-5D index for patients in the US was similar to the reported population norm of 0.81 for the age group 45–54 [41], while a significantly lower index for the CEE population was observed indicating a poorer QoL in CEE patients in this sample. Scores of over 8 on ASAS HI are considered to represent severe disease [42, 43]. In this study, less than one-third of US patients had severe disease, whereas in CEE, half of all patients had severe disease.

Health-related work productivity is generally described in terms of absenteeism (time away from work), presenteeism (extent to which work productivity is impaired while at work), and disability (limit to activities). Previous studies examining the impact of chronic conditions on productivity have estimated that on the job work impairment ranged from a 18–36% decrement in ability to function at work [44]. This study showed comparable results. In terms of disability (activity impairment), this was found to be significantly higher in CEE than the US. Absenteeism was double that of the US in CEE.

### Limitations

This was a non-interventional study, with physicians completing forms on consecutively consulting patients with AS. However, selection bias was possible owing to the fact that physicians surveyed represented a convenience sample and may not be representative of the overall population of physicians treating patients with AS in the US and CEE. Eligible patients were screened and selected by physicians, and it is therefore recognised that patients who were visiting physicians more often were more likely to have been included in the study. Nonetheless, this is reflective of real-world clinical practice, and representative of a consulting population. To minimise these factors, physicians were recruited from a diverse geographical spread and mixed private/public practice. The selection of patients was made based on physicians’ clinical judgement; they might have not always used a radiograph to confirm sacroiliitis at time of diagnosis or completion of the forms or they may have done a radiograph at a different time point. Participating patients were encouraged, but not mandated, to complete all questionnaires, such that base sizes fluctuated across different variables. Finally, it is acknowledged that the study relied on the accuracy of physicians when completing each PRF. To minimise the risk of collecting inaccurate data, PRFs were relatively short and user-friendly with electronic routing and logic applied to ensure no contradictions in responses and, where appropriate, physicians were provided the opportunity of entering “don’t know” if the information was not available.

## Conclusion

This study was novel in providing data on the real-world approach to the diagnosis, treatment, and burden of axSpA and, specifically, AS, in CEE compared with the US. The reported delay in diagnosis and poorer access to biologics may have resulted in the higher disease activity, greater levels of pain, and poorer outcomes as reported by patients with axSpA in CEE. Providing early access to treatment with bDMARDs may improve symptoms and QoL, and increase work productivity and daily activities in patients with AS in CEE countries.

## Supplementary Information

Below is the link to the electronic supplementary material.

Supplementary file1 (61.7 KB)

## Data Availability

All data that support the findings of this study are the intellectual property of Adelphi Real World. All requests for access should be addressed directly to E. Holdsworth at elizabeth.holdsworth@adelphigroup.com.

## Code availability

All analyses carried out in the course of this study used Stata Statistical Software: Release 15 (StataCorp LP, College Station, TX). Further inquiries regarding details of data analysis should be addressed to E. Holdsworth at elizabeth.holdsworth@adelphigroup.com.
