# Correction to: Investigating diagnosis, treatment, and burden of disease in patients with ankylosing spondylitis in Central Eastern Europe and the United States: a real‑world study

**DOI:** 10.1007/s10067-022-06069-3

**Published:** 2022-01-27

**Authors:** T. Korotaeva, O. Dina, E. Holdsworth, L. Fallon, G. Milligan, S. Meakin, L. Wang, R. Vasilescu, J. C. Cappelleri, A. Deodhar

**Affiliations:** 1 Institute of Rheumatology V.A. Nasonova, 115522 Kashirskoe shosse 34‑A, Moscow, Russia; 2 Pfizer Inc, New York, NY USA; 3 Adelphi Real World, Bollington, UK; 4 Pfizer Inc, Kirkland, QC Canada; 5 Pfizer Inc, Groton, CT USA; 6 Pfizer, Brussels, Belgium; 7 Oregon Health & Science University, Portland, OR USA

**Correction to: Clinical Rheumatology (2021) 40:4915–4926**

https://doi.org/10.1007/s10067-021–05864-8

The article “Investigating diagnosis, treatment, and burden of disease in patients with ankylosing spondylitis in Central Eastern Europe and the United States: a real‑world study”, written by T. Korotaeva, O. Dina, E. Holdsworth, L. Fallon, G. Milligan, S. Meakin, L. Wang, R. Vasilescu, J. C. Cappelleri and A. Deodhar, was originally published Online First without Open Access. After publication in volume 40, issue 12, page 4915–4926 the author decided to opt for Open Choice and to make the article an Open Access publication. Therefore, the copyright of the article has been changed to © The Author(s) 2022 and the article is forthwith distributed under the terms of the Creative Commons Attribution 4.0 International License, which permits use, sharing, adaptation, distribution and reproduction in any medium or format, as long as you give appropriate credit to the original author(s) and the source, provide a link to the Creative Commons licence, and indicate if changes were made. The images or other third party material in this article are included in the article’s Creative Commons licence, unless indicated otherwise in a credit line to the material. If material is not included in the article’s Creative Commons licence and your intended use is not permitted by statutory regulation or exceeds the permitted use, you will need to obtain permission directly from the copyright holder. To view a copy of this licence, visit http://creativecommons.org/licenses/by/4.0.

