# Supplementary material for: Investigating diagnosis, treatment, and burden of disease in patients with ankylosing spondylitis in Central Eastern Europe and the United States: a real-world study
Source: Clin Rheumatol. 2021 Jul 28;40(12):4915–26. doi: 10.1007/s10067-021-05864-8 (PMC8794925; doi:10.1007/s10067-021-05864-8)
Supplement: Supplementary file 1 — Supplementary file1 (61.7 KB) [file 10067_2021_5864_MOESM1_ESM.docx]

**SUPPLEMENTARY TABLES**

**Online Resource 1. Outcomes variable list**

| **Outcome variable** | **Physician or patient reported** | **Type of variable** | **Description of variable** |
| --- | --- | --- | --- |
| Age, years | Physician | Continuous | 18-100 |
| Gender | Physician | Categorical (binary) | Male, Female |
| BMI | Physician | Continuous | 15.0-60.0 |
| Ethnicity | Physician | Categorical (multi) | - White/Caucasian - African American/Afro-Caribbean (US) - Native American (US) - Asian-Indian subcontinent - Asian - other - Chinese - Hispanic/Latino (US) - Middle Eastern - Mixed race - Asian - Other |
| Employment status | Physician | Categorical (multi) | - Working full-time - Working part-time - On long-term sick leave - Homemaker/student/retired - Unemployed |
| Smoking status | Physician | Categorical (multi) | - Current smoker - Ex-smoker - Never smoked |
| Patient severity at diagnosis | Physician | Categorical (multi) | - Mild - Moderate - Severe |
| HLA-B27 status | Physician | Categorical (binary) | - Negative or untested - Positive |
| Current concomitant conditions | Physician | Categorical (multi) | - Depression - Anxiety - Rheumatoid arthritis - Psoriatic arthritis - Psoriasis - Crohn’s disease - Uveitis |
| Charlson comorbidity index, mean no. of comorbidities | Physician | Continuous | No min. or max. |
| Current disease features | Physician | Categorical (multi) | - Sacroiliitis identified by x-ray - Sacroiliitis identified by MRI - Spinal fusion - Joint inflammation/stiffness (not spine) - IBP or spinal pain - Back pain for 1-3 months - Back pain for more than 3 months - IBP - Morning stiffness for more than 30 minutes - Alternating buttock pain - Dactylitis - Enthesitis - Tendonitis - Synovitis |
| Time from onset of symptoms to diagnosis (years) | Physician | Time to event | No min. or max. |
| Time from symptom onset to initial consultation (years) | Physician | Time to event | No min. or max. |
| Time from initial consultation to diagnosis (years) | Physician | Time to event | No min. or max. |
| Referring physician | Physician | Categorical (multi) | - Another specialist - Family doctor - Other physician - No-one |
| Diagnosing physician | Physician | Categorical (multi) | - Rheumatologist - Orthopaedic surgeon - Internal medicine - Other |
| Time to referral from previous to current HCP (months) | Physician | Time to event | No min. or max. |
| Reason for delay | Physician | Categorical (multi) | - Waiting for referral to correct HCP - Requiring test to confirm diagnosis - Awaiting test results - Complicated diagnosis - Other condition initially diagnosed - Another condition took precedence - Symptoms not requiring further investigation - Other |
| Time before patient visited HCP (months) | Patient | Time to event | No min. or max. |
| Reason for delay in seeking medical advice | Patient | Categorical (multi) | - Waited for symptoms to resolve unaided - Tied to treat the symptoms myself first - Worried about the diagnosis - Worried about the cost of treatment - Did not think it was anything serious - Thought it was temporary lower back pain - Other reason |
| Time from diagnosis to first bDMARD (years) | Physician | Time to event | No min. or max. |
| Treatments prescribed at diagnosis | Physician | Categorical (multi) | - NSAID - COX-2 inhibitor - Non-opioid analgesic - Opioid analgesic - Oral steroid - Injected steroid - csDMARD - bDMARD - None of the above - Did not know |
| Treatments currently prescribed | Physician | Categorical (multi) | - NSAID - COX-2 inhibitor - Non-opioid analgesic - Opioid analgesic - Oral steroid - Injected steroid - csDMARD - bDMARD - None of the above |
| ASQoL | Patient | PRO tool | 0-18 scale, higher = worse outcome |
| ASAS HI | Patient | PRO tool | 0-17 scale, higher = worse outcome |
| EQ-5D | Patient | PRO tool | 0-1.0 scale, lower = worse outcome |
| BASDAI | Patient | PRO tool | 0-10 scale, higher = worse outcome |
| WPAI | Patient | PRO tool | 0-100% scale, higher = worse outcome |

Abbreviations: ASAS HI, Assessment of Spondyloarthritis International Society Health Index; ASQoL, Ankylosing Spondylitis Quality of Life Questionnaire; BASDAI, Bath Ankylosing Spondylitis Disease Activity Index; bDMARD, biological disease modifying antirheumatic drug; BMI, Body Mass Index; COX-2, Cyclo-oxygenase-2; csDMARD, conventional synthetic disease-modifying antirheumatic drug; EQ-5D, EuroQoL 5D; HCP, healthcare professional; HLA-B27, human leucocyte antigen B27; IBP, inflammatory back pain; MRI, magnetic resonance imaging; NSAID, nonsteroidal anti-inflammatory drug; PRO, patient-reported outcome; SD, standard deviation; US, United States; WPAI, Work Productivity and Activity Impairment

**Online Resource 2. Patient demographic and clinical characteristics, sensitivity analysis including only patients whose physician stated they had sacroiliitis identified by x-ray at diagnosis**

|  | **US** | **CEE** | **p-value** |
| --- | --- | --- | --- |
| **Age, years, n** | 272 | 625 | Ns |
| Mean (SD) | 44.8 (13.8) | 45.3 (12.0) |  |
| Median (IQR) | 44.0 (34.5-54.5) | 45.0 (37.0-54.0) |  |
| **Gender, n (%)** | 272 | 625 | Ns |
| Male | 202 (74.3%) | 467 (74.7%) |  |
| Female | 70 (25.7%) | 158 (25.3%) |  |
| **BMI, n** | 272 | 625 | 0.003 |
| Mean (SD) | 27.0 (4.7) | 26.0 (4.2) |  |
| Median (IQR) | 26.3 (24.0-28.9) | 25.6 (23.3-28.4) |  |
| **Ethnicity, n (%)** | 272 | 625 | <0.001 |
| White/Caucasian | 216 (79.4%) | 607 (97.1%) |  |
| African-American/Afro-Caribbean | 23 (8.5%) | 1 (0.2%) |  |
| Native American | 1 (0.4%) | 0 (0.0%) |  |
| Asian-Indian Subcontinent | 5 (1.8%) | 0 (0.0%) |  |
| Asian–other | 7 (2.6%) | 0 (0.0%) |  |
| Chinese | 2 (0.7%) | 0 (0.0%) |  |
| Hispanic/Latino | 14 (5.1%) | 0 (0.0%) |  |
| Middle Eastern | 2 (0.7%) | 2 (0.3%) |  |
| Mixed race | 1 (0.4%) | 2 (0.3%) |  |
| Asian | 0 (0.0%) | 3 (0.5%) |  |
| Other | 1 (0.4%) | 10 (1.6%) |  |
| **Employment status, n (%)** | 269 | 608 | <0.001 |
| Working full-time | 193 (71.7%) | 338 (55.6%) |  |
| Working part-time | 22 (8.2%) | 92 (15.1%) |  |
| On long-term sick leave | 3 (1.1%) | 22 (3.6%) |  |
| Homemaker/Student/Retired | 37 (13.8%) | 120 (19.7%) |  |
| Unemployed | 14 (5.2%) | 36 (5.9%) |  |
| **Smoking status, n (%)** | 263 | 553 |  |
| Current smoker | 28 (10.6%) | 123 (22.2%) | <0.001 |
| Ex-smoker | 58 (22.1%) | 159 (28.8%) | Ns |
| Never smoked | 177 (67.3%) | 271 (49.0%) | <0.001 |
| **Patient severity at diagnosis, n (%)^a^** | 238 | 582 | Ns |
| Mild | 13 (5.5%) | 38 (6.5%) |  |
| Moderate | 129 (54.2%) | 297 (51.0%) |  |
| Severe | 96 (40.3%) | 247 (42.4%) |  |
| **HLA-B27 status, n (%)** | 272 | 625 | Ns |
| Negative or untested | 62 (22.8%) | 158 (25.3%) |  |
| HLA-B27 positive | 210 (77.2%) | 467 (74.7%) |  |
| **Current concomitant conditions, n (%)** | 272 | 625 |  |
| Depression | 31 (11.4%) | 18 (2.9%) | <0.001 |
| Anxiety | 24 (8.8%) | 35 (5.6%) | Ns |
| Rheumatoid arthritis | 1 (0.4%) | 15 (2.4%) | Ns |
| Psoriatic arthritis | 2 (0.7%) | 12 (1.9%) | Ns |
| Psoriasis | 6 (2.2%) | 23 (3.7%) | Ns |
| Crohn’s disease | 11 (4.0%) | 6 (1.0%) | 0.005 |
| Uveitis | 23 (8.5%) | 44 (7.0%) | Ns |
| **Charlson comorbidity index** | 272 | 625 | <0.001 |
| Mean (SD) | 0.1 (0.4) | 0.3 (0.8) |  |
| Median (IQR) | 0.0 (0.0-0.0) | 0.0 (0.0-0.0) |  |
| **Current symptoms, n (%)** | 272 | 625 |  |
| Sacroiliitis identified by x-ray | 117 (43.0%) | 419 (67.0%) | <0.001 |
| Sacroiliitis identified by MRI | 43 (15.8%) | 143 (22.9%) | 0.016 |
| Spinal fusion | 38 (14.0%) | 94 (15.0%) | Ns |
| Joint inflammation/stiffness (not spine) | 52 (19.1%) | 115 (18.4%) | Ns |
| IBP or spinal pain | 102 (37.5%) | 350 (56.0%) | <0.001 |
| Back pain for 1-3 months | 3 (1.1%) | 38 (6.1%) | <0.001 |
| Back pain for more than 3 months | 37 (13.6%) | 161 (25.8%) | <0.001 |
| Morning stiffness for more than 30 minutes | 99 (36.4%) | 209 (33.4%) | Ns |
| Alternating buttock pain | 15 (5.5%) | 73 (11.7%) | 0.005 |
| Dactylitis | 7 (2.6%) | 13 (2.1%) | Ns |
| Enthesitis | 18 (6.6%) | 34 (5.4%) | Ns |
| Tendonitis | 10 (3.7%) | 14 (2.2%) | Ns |
| Synovitis | 13 (4.8%) | 14 (2.2%) | Ns |
| None of the above | 53 (19.5%) | 60 (9.6%) | <0.001 |
| **Symptoms at diagnosis, n (%)** | 272 | 625 |  |
| Sacroiliitis identified by x-ray | 272 (100.0%) | 625 (100.0%) | <0.001 |
| Sacroiliitis identified by MRI | 84 (30.9%) | 271 (43.4%) | <0.001 |
| Spinal fusion | 52 (19.1%) | 125 (20.0%) | Ns |
| Joint inflammation/stiffness (not spine) | 127 (46.7%) | 258 (41.3%) | Ns |
| IBP or spinal pain | 230 (84.6%) | 583 (93.3%) | <0.001 |
| Back pain for 1-3 months | 17 (6.3%) | 75 (12.0%) | 0.008 |
| Back pain for more than 3 months | 138 (50.7%) | 377 (60.3%) | 0.008 |
| Morning stiffness for more than 30 minutes | 203 (74.6%) | 414 (66.2%) | 0.015 |
| Alternating buttock pain | 51 (18.8%) | 198 (31.7%) | <0.001 |
| Dactylitis | 16 (5.9%) | 37 (5.9%) | Ns |
| Enthesitis | 53 (19.5%) | 120 (19.2%) | Ns |
| Tendonitis | 31 (11.4%) | 52 (8.3%) | Ns |
| Synovitis | 35 (12.9%) | 98 (15.7%) | Ns |

^a^Severity adjudged by physician’s subjective opinion

Abbreviations: Ns, Non-significant; BMI, Body Mass Index; CEE, Central Eastern European countries; HLA-B27, human leucocyte antigen B27; IBP, inflammatory back pain; IQR, interquartile range; MRI, magnetic resonance imaging; SD, standard deviation; US, United States

**Online Resource 3. Patient journey from initial symptoms to diagnosis of AS (physician-reported), sensitivity analysis including only patients whose physician stated they had sacroiliitis identified by x-ray at diagnosis**

|  | **US** | **CEE** | **P-value** |
| --- | --- | --- | --- |
| **Time from symptom onset to diagnosis (years)** |  |  |  |
| N | 168 | 548 |  |
| Mean | 2.8 | 4.6 | 0.004 |
| SD | 6.3 | 6.9 |  |
| **Time from symptom onset to initial consultation (years)** |  |  |  |
| N | 147 | 519 |  |
| Mean | 1.4 | 2.7 | 0.006 |
| SD | 4.3 | 5.1 |  |
| **Time from initial consultation to diagnosis (years)** |  |  |  |
| N | 165 | 534 |  |
| Mean | 0.5 | 1.6 | 0.002 |
| SD | 1.1 | 4.4 |  |

Abbreviations: AS, Ankylosing spondylitis; CEE, Central Eastern European countries; SD, standard deviation; US, United States

**Online Resource 4. Patient journey to current rheumatologist (physician-reported), sensitivity analysis including only patients whose physician stated they had sacroiliitis identified by x-ray at diagnosis**

|  | **US** | **CEE** | **P-value** |
| --- | --- | --- | --- |
| **Referring physician, % (SE)^a^** |  |  |  |
| Another specialist | 43.9 (3.1) | 32.7 (1.9) | <0.001 |
| Family doctor | 30.0 (2.8) | 34.5 (1.9) |  |
| Other physician | 3.3 (1.1) | 17.6 (1.6) |  |
| No one | 22.8 (2.6) | 15.3 (1.5) |  |
| **Diagnosing physician, % (SE)^a^** |  |  |  |
| Rheumatologist | 91.4 (1.8) | 93.6 (1.0) | Ns |
| Orthopedic surgeon | 3.2 (1.1) | 1.3 (0.4) |  |
| Internal medicine | 2.1 (0.9) | 1.1 (0.4) |  |
| Other | 3.3 (1.1) | 4.1 (0.8) |  |
| **Time to referral from previous HCP to current rheumatologist (months), n** |  |  |  |
| Mean^b^ | 4.6 | 13.8 | 0.014 |
| SE | 3.18 | 1.88 |  |
| **Reason for delay, % (SE)^a^** |  |  |  |
| Waiting for referral to correct HCP | 27.5 (6.5) | 19.8 (2.6) | Ns |
| Needed test conducting to confirm diagnosis | 33.2 (6.8) | 27.6 (2.9) | Ns |
| Waiting for test results | 22.3 (5.9) | 23.0 (2.7) | Ns |
| Complicated diagnosis | 19.1 (5.6) | 11.4 (2.0) | Ns |
| Other condition initially diagnosed - What condition? | 12.7 (4.8) | 36.0 (3.1) | 0.003 |
| Another condition took precedence - What condition? | 2.2 (2.2) | 6.3 (1.6) | Ns |
| Symptoms not prominent enough for further investigation | 10.7 (4.5) | 10.1 (2.0) | Ns |
| Other | 12.3 (4.8) | 7.2 (1.7) | Ns |
| Don’t know | 17.2 (5.5) | 12.6 (2.1) | Ns |

^a^Least square means (percentages), standard errors and p-values derived from logistic regressions with additional covariates: Age, sex, BMI and Charlson comorbidity index

^b^Predicted means, standard errors and p-value are from an ordinary least squares regression with additional covariates: Age, sex, BMI and Charlson comorbidity index

Abbreviations: Ns, Non-significant; BMI, Body Mass Index; CEE, Central Eastern European countries; HCP, healthcare professional; SE, standard error; US, United States

**Online Resource 5. Patient self-reported time to first consultation, sensitivity analysis including only patients whose physician stated they had sacroiliitis identified by x-ray at diagnosis**

|  | **US** | **CEE** | **P-value** |
| --- | --- | --- | --- |
| **Time before patient visited HCP (months)** |  |  |  |
| Mean^a^ | 39.5 | 27.1 | Ns |
| SE | 6.0 | 2.8 |  |
| **Reason for delay in seeking medical advice, mean % (SD)** |  |  |  |
| Waited for symptoms to resolve unaided | 56.0 (4.1) | 49.0 (2.1) | Ns |
| Tried to treat the symptoms myself first | 31.2 (3.9) | 31.2 (1.9) | Ns |
| Worried about the diagnosis | 16.0 (3.0) | 10.6 (1.3) | Ns |
| Worried about the cost of treatment | 12.5 (2.9) | 1.9 (0.6) | <0.001 |
| Did not think it was anything serious | 37.5 (4.0) | 27.1 (1.9) | 0.015 |
| Thought it was temporary lower back pain | 37.4 (4.1) | 39.9 (2.1) | Ns |
| Other reason | 10.6 (2.6) | 7.9 (1.1) | Ns |
| **Time from diagnosis to first bDMARD (years), n** |  |  |  |
| Mean | 2.9 | 4.1 |  |
| SD | 5.6 | 5.9 |  |

^a^Least square means, standard error, and p-value are from an ordinary least squares regression with additional covariates: Age, sex, BMI and Charlson comorbidity index

Abbreviations: Ns, Non-significant; bDMARD, biological disease modifying antirheumatic drug; BMI, Body Mass Index; CEE, Central Eastern European countries; HCP, healthcare professional; SD, standard deviation; SE, standard error; US, United States

**Online Resource 6. Treatment patterns, sensitivity analysis including only patients whose physician stated they had sacroiliitis identified by x-ray at diagnosis**

|  | **US** | **CEE** | **P-values** |
| --- | --- | --- | --- |
| **Treatments prescribed at diagnosis, % (SE)**^a^ |  |  |  |
| NSAID | 84.4 (2.2) | 84.1 (1.5) | Ns |
| COX-2 inhibitor | 7.3 (1.6) | 26.9 (1.8) | <0.001 |
| Non-opioid analgesic | 8.2 (1.7) | 14.0 (1.3) | 0.017 |
| Opioid analgesic | 9.3 (1.8) | 3.1 (0.7) | <0.001 |
| Oral steroid | 14.1 (2.2) | 13.1 (1.3) | Ns |
| Injected steroid | 5.3 (1.4) | 18.4 (1.5) | <0.001 |
| csDMARD | 24.0 (2.6) | 45.5 (2.0) | <0.001 |
| bDMARD | 30.1 (2.8) | 12.2 (1.3) | <0.001 |
| None of the above | 1.5 (0.8) | 0.8 (0.4) | Ns |
| Don’t know | 6.1 (1.5) | 1.5 (0.5) | <0.001 |
| **Treatments currently prescribed, % (SE)^a^** |  |  |  |
| NSAID | 46.8 (3.0) | 61.4 (1.9) | <0.001 |
| COX-2 inhibitor | 10.4 (1.9) | 29.1 (1.8) | <0.001 |
| Non-opioid analgesic | 4.2 (1.3) | 7.2 (1.0) | Ns |
| Opioid analgesic | 8.1 (1.7) | 1.7 (0.5) | <0.001 |
| Oral steroid | 5.2 (1.4) | 8.2 (1.1) | Ns |
| Injected steroid | 3.5 (1.1) | 4.9 (0.9) | Ns |
| csDMARD | 19.2 (2.4) | 42.3 (2.0) | <0.001 |
| bDMARD | 75.9 (2.6) | 31.9 (1.9) | <0.001 |
| None of the above | 1.2 (0.7) | 1.1 (0.5) | Ns |

^a^Least square means (percentages), standard errors, and p-values are from logistic regressions with additional covariates: Age, sex, BMI and Charlson comorbidity index

Abbreviations: Ns, Non-significant; bDMARD, biological disease modifying antirheumatic drug; BMI, Body Mass Index; CEE, Central Eastern European countries; COX-2, Cyclo-oxygenase-2; csDMARD, conventional synthetic disease-modifying antirheumatic drug; HCP, healthcare professional; NSAID, nonsteroidal anti-inflammatory drug; SE, standard error; US, United States

**Online Resource 7. ASQoL, ASAS HI, EQ-5D Index, BASDAI, and** **WPAI mean scores^a^, sensitivity analysis including only patients whose physician stated they had sacroiliitis identified by x-ray at diagnosis**

|  | **US** | **CEE** | **P-value** |
| --- | --- | --- | --- |
| **ASQoL** |  |  |  |
| Mean^a^ | 5.4 | 8.4 | <0.001 |
| SE | 0.48 | 0.24 |  |
|  |  |  |  |
| ***Descriptive*** |  |  |  |
| N | 139 | 557 |  |
| Mean | 5.4 | 8.4 |  |
| SD | 5.4 | 5.8 |  |
| **ASAS HI** |  |  |  |
| Mean^a^ | 5.2 | 8.1 | <0.001 |
| SE | 0.4 | 0.2 |  |
|  |  |  |  |
| ***Descriptive*** |  |  |  |
| N | 139 | 535 |  |
| Mean | 5.2 | 8.1 |  |
| SD | 4.6 | 4.8 |  |
| **EQ-5D Index** |  |  |  |
| Mean^a^ | 0.8 | 0.7 | <0.001 |
| SE | 0.01 | 0.01 |  |
|  |  |  |  |
| ***Descriptive*** |  |  |  |
| N | 141 | 558 |  |
| Mean | 0.8 | 0.7 |  |
| SD | 0.2 | 0.2 |  |
| **BASDAI** |  |  |  |
| Mean^a^ | 3.0 | 4.2 | <0.001 |
| SE | 0.18 | 0.09 |  |
|  |  |  |  |
| ***Descriptive*** |  |  |  |
| N | 140 | 551 |  |
| Mean | 3.0 | 4.2 |  |
| SD | 2.4 | 2.1 |  |
| **WPAI: Percent overall work impairment due to problem** |  |  |  |
| Mean^a^ | 19.4 | 33.3 | <0.001 |
| SE | 2.42 | 1.36 |  |
|  |  |  |  |
| ***Descriptive*** |  |  |  |
| N | 84 | 261 |  |
| Mean | 19.1 | 33.4 |  |
| SD | 19.3 | 22.6 |  |
| **WPAI: Percent work time missed due to problem** |  |  |  |
| Mean^a^ | 7.2 | 10.7 | Ns |
| SE | 2.6 | 1.4 |  |
|  |  |  |  |
| ***Descriptive*** |  |  |  |
| N | 88 | 315 |  |
| Mean | 7.3 | 10.7 |  |
| SD | 22.3 | 24.8 |  |
| **WPAI: Percent impairment while working due to problem** |  |  |  |
| Mean^a^ | 17.2 | 31.0 | <0.001 |
| SE | 2.1 | 1.2 |  |
|  |  |  |  |
| ***Descriptive*** |  |  |  |
| N | 91 | 288 |  |
| Mean | 16.9 | 31.1 |  |
| SD | 17.1 | 20.9 |  |
| **WPAI: Percent activity impairment due to problem** |  |  |  |
| Mean^a^ | 28.4 | 41.2 | <0.001 |
| SE | 2.0 | 1.0 |  |
|  |  |  |  |
| ***Descriptive*** |  |  |  |
| N | 138 | 543 |  |
| Mean | 28.5 | 41.2 |  |
| SD | 25.1 | 23.2 |  |

^a^Least square means and p-value are from an ordinary least squares regression with additional covariates: Age, sex, BMI and Charlson comorbidity index

Abbreviations: Ns, Non-significant; ASAS HI, Assessment of Spondyloarthritis International Society Health Index; ASQoL, Ankylosing Spondylitis Quality of Life Questionnaire; BASDAI, Bath Ankylosing Spondylitis Disease Activity Index; BMI, Body Mass Index; CEE, Central Eastern European countries; EQ-5D, EuroQoL 5D; SD, standard deviation; SE, standard error; US, United States; WPAI, Work Productivity and Activity Impairment
